# Streptococcal Endo-β-*N*-Acetylglucosaminidase Suppresses Antibody-Mediated Inflammation *In Vivo*

**DOI:** 10.3389/fimmu.2018.01623

**Published:** 2018-07-16

**Authors:** Kutty Selva Nandakumar, Mattias Collin, Kaisa E. Happonen, Susanna L. Lundström, Allyson M. Croxford, Bingze Xu, Roman A. Zubarev, Merrill J. Rowley, Anna M. Blom, Christian Kjellman, Rikard Holmdahl

**Affiliations:** ^1^School of Pharmaceutical Sciences, Southern Medical University, Guangzhou, China; ^2^Medical Inflammation Research, Department of Medical Biochemistry and Biophysics, Karolinska Institute, Stockholm, Sweden; ^3^Division of Infection Medicine, Department of Clinical Sciences, Lund University, Lund, Sweden; ^4^Department of Translational Medicine, Lund University, Lund, Sweden; ^5^Molecular Neurobiology Laboratory, Salk Institute for Biological Studies, La Jolla, CA, United States; ^6^Division of Physiological Chemistry I, Department of Medical Biochemistry and Biophysics, Karolinska Institute, Stockholm, Sweden; ^7^Department of Biochemistry and Molecular Biology, Monash University, Clayton, VIC, Australia; ^8^Hansa Medical AB, Lund, Sweden

**Keywords:** endoglycosidase, arthritis, rheumatoid, glycosylation, mouse models, complement, immunoglobulin G, immunohistochemistry

## Abstract

Endo-β-*N*-acetylglucosaminidase (EndoS) is a family 18 glycosyl hydrolase secreted by *Streptococcus pyogenes*. Recombinant EndoS hydrolyzes the β-1,4-di-*N*-acetylchitobiose core of the N-linked complex type glycan on the asparagine 297 of the γ-chains of IgG. Here, we report that EndoS and IgG hydrolyzed by EndoS induced suppression of local immune complex (IC)-mediated arthritis. A small amount (1 µg given i.v. to a mouse) of EndoS was sufficient to inhibit IgG-mediated arthritis in mice. The presence of EndoS disturbed larger IC lattice formation both *in vitro* and *in vivo*, as visualized with anti-C3b staining. Neither complement binding *in vitro* nor antigen-antibody binding *per se* were affected. Thus, EndoS could potentially be used for treating patients with IC-mediated pathology.

## Introduction

Endoglycosidases form a group (E.C.3.2 subclass) of enzymes that hydrolyze non-terminal glycosidic bonds in oligosaccharides or polysaccharides. Endo-β-*N*-acetylglucosaminidase (EndoS) is a member of the GlcNAc polymer hydrolyzing glycosyl hydrolases of family 18 (FGH18) secreted by group A β-hemolytic *Streptococcus pyogenes*. It exclusively hydrolyzes the β-1,4-di-*N*-acetylchitobiose core of the asparagine-linked complex-type glycan on Asn-297 of the γ-chains of IgG (1). The crystal structure of EndoS revealed that it contains five distinct protein domains (glycosidase, leucine-rich repeat, hybrid Ig, carbohydrate-binding module, and three-helix bundle domains) (2) and concerted conformational changes in both the enzyme and substrate are required for subsequent antibody deglycosylation (2, 3). Recently, functionally and structurally related enzymes have been identified in certain serotypes of *S. pyogenes, Streptococcus dysgalactiae*, and *Corynebacterium pseudotuberculosis* (4–6).

Glycosylation is an important post-translational modification affecting the structure and biological properties of proteins. Subtle changes in IgG N-glycome could alter Fc conformation with dramatic consequences for IgG effector functions (7). Extensive non-covalent interactions between the carbohydrate and the protein moiety in the IgG-Fc region result in reciprocal influences on conformation (8). NMR studies suggested a significant role for Fc-glycan dynamics in Fc receptor (FcR) interactions (9). The minimal oligosaccharide structure in IgG is a hexasaccharide (GlcNAc2Man3GlcNAc) with variable sugar residues attached resulting in the generation of many different glycoforms. Altered IgG glycoforms lacking terminal sialic acid and galactose residues were identified to be more common on IgG from RA patients (10). Differential sialylation was reported to regulate the inflammatory property of IgG (11). Furthermore, a single molecule Förster resonance energy transfer study has shown that Fc deglycosylation resulting from EndoS treatment led to wide changes in Fc conformation, which enhanced its flexibility (12).

Antibodies such as anti-citrullinated protein antibodies (ACPA), rheumatoid factors (RF), anti-type II collagen antibodies, and immune complexes (ICs) are prevalent in RA. ACPA and RF also precede disease development (13, 14). IC-mediated pathology is evident in several autoimmune diseases. Importantly, the pathogenic effect of circulating IC was shown to be dependent on their size and composition (15). Both antigen-driven (soluble and target tissue-bound) and RF containing ICs present in RA patients are of intermediate (6S–19S) to large (22S–30S) size. The larger ICs containing RF (>22S) are implicated in extra-articular manifestations in RA (16). Furthermore, CII containing ICs from RA synovial fluid were shown to induce production of inflammatory cytokines (TNF-α, IL-1β, and IL-8) from peripheral blood mononuclear cells *via* FcγRIIA (17).

Antibodies from RA patients upon passive transfer induced arthritis in mice (18, 19). The effector phase of arthritis is optimally studied using the collagen antibody-induced arthritis (CAIA) model, which is induced by anti-CII IgG mAbs (20). This model exhibits features of bone and cartilage erosion, major infiltration of granulocytes, and deposition of IgG and complement factors on the cartilage surface. CAIA is characteristic of RA and is dependent on complement, FcγRs, TNF-α, IL-1β, and neutrophils as well as macrophages (20). Furthermore, it should be mentioned that human intravenous immunoglobulins (hIVIg) were earlier used at very high concentrations (1 g/kg) and 1 h before arthritogenic serum transfer to attenuate joint inflammation and the anti-inflammatory property of IgG was shown to be mainly due to Fc sialylation (11). Interestingly, removal of the N-linked glycan by EndoS treatment abrogated the pathogenic potential of antibodies (21–23) and abolished all the pro-inflammatory properties of IC from SLE patients (24). In addition, EndoS-hydrolyzed IgG ameliorated several antibody-mediated diseases in mice, including arthritis (22). Attenuation of inflammation was reported to be dependent on IgG_1_ and IgG_2b_ subclasses but the mechanisms were not clarified. EndoS hydrolyzes all the IgG subclasses in man and mice but no effects on the other isotypes have been detected under physiological conditions. Here, we demonstrate suppression of inflammatory arthritis by minimal amounts of EndoS, an effect which is dominant and mediated through disturbances in the formation of ICs on the cartilage surface.

## Materials and Methods

### Mice

The B10.Q founder mice were obtained originally from Professor Jan Klein (Tubingen, Germany) and have been maintained in our laboratory for more than 20 years. The BALB/c founder mice were obtained from Jackson laboratories (Bar Harbor, ME, USA). (BALB/c × B10.Q) F1 mice, short named as QB, were bred in the Medical Inflammation Research animal house facility in Lund and Stockholm. For *in vivo* experiments shown in Figure 2, arthritis was induced in male BALB/c mice obtained from Harlan, Denmark. The animals were kept in a specific pathogen free (Felasa II) animal facility with a climate controlled environment having 12-h light/dark cycles in polystyrene cages containing wood shavings and were fed standard rodent chow and water *ad libitum*. Two- to four-months-old male mice were used in all the experiments, with the experimental groups matched for age, mixed in cages, and run blindly. Splenectomy or sham operation was done under isoflurane anesthesia. Two- to three-days-old pups were used for immunohistochemical studies. Local animal welfare authorities approved the animal experiments.

### Antibodies

The CII-specific hybridomas (M2139, M284, CIIC1, CIIC2, CB20, and UL1) were generated and characterized as described (26–28). ACC4 antibody recognizes the citrullinated CII epitope, C1 (29). A mouse anti-trinitrophenol (anti-TNP) antibody-producing hybridoma (Hy2.15) was a gift from Georges Köhler (30). A mouse monoclonal hybridoma recognizing trophozoite antigens (2B5.3; CRL-1960) and human HLA-DR α-chain specific antibody clone (L243) were obtained from ATCC (Rockville, MD, USA). M284 and CB20 mAbs (31) bind CII epitopes, J1 and C1, respectively. Mouse intravenous immunoglobulins (mIVIg; Equitech-Bio Inc.) and hIVIg (Octagam, Octapharma AB) were used. All the hybridomas were cultured in ultra-low bovine IgG-containing DMEM Glutamax-I culture medium (Gibco BRL, Invitrogen AB, Sweden) with 100 mg/l of kanamycin monosulfate (Sigma, St. Louis, MO, USA). MAbs were generated on a large scale as culture supernatant using Integra cell line 1000 (CL-1000) flasks (Integra Biosciences, Switzerland). Antibodies were purified using γ-bind plus affinity gel matrix (GE Healthcare, Sweden) and the Äkta purification system (GE Healthcare, Sweden) as described (20). Briefly, culture supernatants were centrifuged at 12,500 rpm for 30 min, filtered, and degassed before applying to the gel matrix. The gel was washed extensively and the antibodies were eluted using acetic acid buffer at pH 3.0 and neutralized with 1 M Tris–HCl, pH 9.0. The peak fractions were pooled and dialyzed extensively against PBS, pH 7.0 with or without azide. The IgG content was determined by freeze drying. The antibody solutions were sterilized using 0.2-µm syringe filters (Dynagard, Spectrum Laboratories, CA, USA), aliquoted, and stored at −70°C.

### EndoS Hydrolysis of IgG

IgGs (CIIC1, M2139, M284, CIIC2, UL1, CB20, ACC4, Hy2.15, L243, CRL-1960, mIVIg and hIVIg) were hydrolyzed with recombinant EndoS fused to GST (GST-EndoS) as previously described (1). Five micrograms of GST-EndoS in PBS were added per milligram of mAb followed by incubation for 16 h at 37°C. GST-EndoS was removed by three serial passages over Glutathione-Sepharose 4B columns with a 1,000-fold overcapacity of GST-binding (GE Healthcare, Uppsala, Sweden). SDS-PAGE and *Lens culinaris* agglutinin (LCA) lectin blotting were used to assess the purity and efficacy of EndoS cleavage. Briefly, 2 µg of EndoS hydrolyzed and unhydrolyzed IgG were separated on 10% SDS-PAGE followed by staining with PageBlue protein stain (Thermo Fisher Scientific), or blotted to PVDF using TransBlot Turbo transfer packs and apparatus (Bio-Rad, Hercules, CA, USA). Membranes were blocked with 10 mM HEPES (pH 7.5) with 0.15 M NaCl, 0.01 mM MnCl_2_, 0.1 mM CaCl_2_, and 0.1% Tween-20 (HBST) and incubated with 1 µg/ml of biotinylated LCA lectin (Vector Laboratories, Burlingame, CA, USA). After washing in HBST, membranes were incubated with 50 ng/ml of peroxidase-labeled streptavidin (Vector Laboratories), and developed using Super Signal West Pico Chemiluminescent Substrate (Thermo Fisher Scientific) and a ChemiDoc XRS imaging system (Bio-Rad). The DeGlycIt treatment was performed following the manufacturer’s instructions (Genovis AB, Lund, Sweden). Briefly, antibodies were added to a column with immobilized EndoS, the column was incubated for 30 min under rotation, and the deglycosylated antibodies were eluted by centrifugation.

### Glycopeptide Identification

Endo-β-*N*-acetylglucosaminidase hydrolyzed or unhydrolyzed antibodies (15 µg) were trypsin digested similar to what has been previously described (32). Samples were analyzed using a reversed phase liquid chromatography system (Easy-nLC, Proxeon) connected to a Velos Orbitrap mass spectrometer (Thermo Fisher Scientific). The MS was operated in positive mode, and the survey MS scan in the range of *m/z* 300–2,000 was obtained at a resolution of 60,000. MS/MS was performed using CID and ETD fragmentation. IgG Fc glycopeptides were identified in LC-MS/MS datasets by their characteristic retention times and accurate monoisotopic masses (within <10 ppm from the theoretical values) of doubly and triply charged ions from M2139: EDYNSTIR, CIIC1, and L243: EDYNSTLR as well as Hy2.15: EEQFNSTFR, respectively. Protein identity was confirmed using MASCOT search engine (V.2.3.2) using IPI mouse concatenated database. Search parameters were as follows: MS mass error tolerance at 10 ppm, MS/MS mass accuracy at 0.5 Da, tryptic digestion with a maximum of two missed-cleavages, carbamidomethylation of cysteine as a fixed modification, asparagine and glutamine deamidation, methionine oxidation as well as N-glycosylation (HexNAc[n]dHex[n]Hex[n]) as variable modifications.

### Rabbit IgG Cleavage Assay

This *in vitro* assay made use of rabbit IgG as a reporter for EndoS activity in combination with another streptococcal enzyme, IgG-degrading enzyme of *Streptococcus pyogenes* (IdeS). IdeS has the ability to efficiently cleave human (33) and rabbit IgG (34) while leaving mouse IgG_1_ and IgG_2b_ intact (35). After IdeS treatment, deglycosylated rabbit Fc-fragment was possible to distinguish from the non-deglycosylated part using SDS-PAGE (i.e., a 25-kDa fragment with and without glycosylation). The reactions contained 5 µg of the EndoS-treated mouse antibodies and 5 µg of rabbit gamma globulin (Jackson ImmunoResearch) and were incubated at 37°C for 60 min during which potentially remaining EndoS will deglycosylate rabbit IgG, the reporter in this assay. After this incubation, IdeS (1 µg) was added and the reaction was allowed to continue for an additional 45 min. SDS loading buffer was added, and one-third of the reaction mixture was separated on SDS-PAGE (4–20% TGX, Bio-Rad), stained, and visualized.

### Particle Size Measurement Using Dynamic Light Scattering (DLS)

To determine the size of ICs by EndoS-hydrolyzed IgGs we used DLS. Briefly, CII purified from rat chondrosarcoma dissolved in 0.1 M acetic acid at 5 mg/ml was diluted further in PBS (1 mg/ml) and anti-CII mAb (high affinity M2139 or low affinity CB20; 1 mg/ml) in PBS were mixed together at 1:1 ratio and incubated for 30 min at 37°C, followed by addition of either unhydrolyzed (Hy2.15) or EndoS-hydrolyzed (Hy2.15H) anti-hapten IgG at the ratio 1:1:1. Twenty microliters of this mixture were loaded on to the capillary tube in the DLS instrument (Precision Detectors Inc., Bellingham, MA, USA). The particle sizes expressed as the apparent *Z*-average (or intensity-weighted) hydrodynamic diameter (dH) and polydispersity index, which provides information on the deviation from monodispersity were measured (36) and compared between the groups.

### Surface Plasmon Resonance (SPR) Analysis

Surface Plasmon Resonance (Biacore 2000; Biacore) analysis was performed using the standard procedure (37). Briefly, CII was immobilized on the surface of CM5 sensor chips. EndoS-hydrolyzed and unhydrolyzed IgGs were injected at different concentrations through flow cells in the running buffer (10 mM HEPES, pH 7.4, 150 mM NaCl, 3.4 mM EDTA, and 0.005% surfactant P20) at a flow rate of 30 µl/min. Antibodies were injected for 3 min and dissociation of bound molecules was observed for 7 min. Background binding to control flow cells was subtracted automatically. The chips were regenerated using pulse injection of 100% ethylene glycol followed by 2 M NaCl and 100 mM HCl.

### Collagen Antibody-Induced Arthritis

Endo-β-*N*-acetylglucosaminidase-hydrolyzed and unhydrolyzed, two or four arthritogenic mAb combinations were studied: M2139 (γ2b), CIIC1 (γ2a), CIIC2 (γ2b), and UL1 (γ2b) bind to triple helical J1 (MP*GERGAAGIAGPK—P* indicates hydroxyproline), C1^I^ (GARGLTGRO) (38), D3 (RGAQGPOGATGF), and U1 (GLVGPRGERGF) CII epitopes, respectively. The cocktail of the mAbs (9 or 4 mg/mouse) was prepared by mixing equal concentrations of each of the sterile filtered antibody solutions. Mice were injected i.v. with 250–500 µl solution. All the mice received LPS (25 µg/mice/i.p.) at day 5 as described earlier (39). CAIA experiment shown in Figure 2 was done as follows: mice were administered with 2 mg/mouse of CII-specific mAb cocktail (MD Biosciences) i.p. on day 0 followed by 50 µg/mouse of LPS (*E. coli* 055:B5) on day 5. The animals were divided into different groups (*n* = 8–10 mice/group), and different substances were administered i.v. on day 0 and the arthritic score of the animals were followed between days 0 and 14. Mice were examined daily for development of arthritis. Scoring of the inflammation was done blindly using a scoring system based on the number of inflamed joints in each paw, inflammation being defined by swelling and redness. In all the arthritis experiments, the maximum score given was 15/paw, 60 for all 4 paws (25), except in Figure 2, where arthritis was evaluated using a scoring scale of 0–16 (25).

### Histological Preparations

Paws were dissected from each group of mice (3–4 mice/group), fixed in 4% phosphate buffered paraformaldehyde solution (pH 7.0) for 24 h, decalcified for 3–4 weeks in a solution containing EDTA, polyvinylpyrrolidone, and Tris–HCl, pH 6.95 followed by dehydration and embedding in paraffin. Sections of 6 µm were stained with hematoxylin-eosin to determine cellular infiltrations and, bone and cartilage morphology. Joint sections were scored as follows: score 0, normal joints. Score 1, mild synovitis with hyperplastic synovial membrane and small focal infiltration of inflammatory cells, increased numbers of vessels in the synovium and a villous formation of synovium but without any bone or cartilage erosions. Score 2, moderate synovitis with pannus formation, bone and cartilage erosions limited to discrete foci and undisturbed joint architecture. Score 3, severe pannus formation with extensive erosions of bone and cartilage with disrupted joint architecture.

For immunohistochemistry, 2–3 days old QB pups (3–4 mice/group) were injected with 1 mg each of a unhydrolyzed antibody cocktail containing M2139 + CIIC2 + UL1 antibodies, EndoS-hydrolyzed IgG (M2139H + CIIC2H + UL1H), or a mixture of unhydrolyzed and EndoS-hydrolyzed IgG at 1:1 ratio. Twenty-four hours later, mice were sacrificed, and paw samples were snap frozen in OCT compound using cold isopentane and dry ice. Sections of 6 µm were stained with biotinylated anti-kappa (clone: 187.1) or goat anti-mouse anti-C3c antibodies (Nordic Immunological Laboratories, Tilburg, The Netherlands). Extravidin-peroxidase and diaminobenzidine were used for detection. CIIC1 mAb was excluded from this cocktail because of their inability to activate complement after binding to CII. Histology scoring of joint sections after anti-C3c antibodies was done as follows: 0, no staining; 1, staining only at sub-chondral bone junction area; 2, weak; and 3, strong staining uniformly on the cartilage surface.

### Complement Activation and RF-Like Activity of CII-Binding Antibodies

Microtiter plates were coated with 3 µg/ml M2139 or CIIC1 alone or in combination with 3, 6, and 12 µg/ml of EndoS-hydrolyzed or unhydrolyzed Hy2.15, L243, M2139, and CIIC1 IgG in 75 mM sodium carbonate buffer pH 9.6 overnight at 4°C. Plates were washed between each step with 50 mM Tris–HCl, 150 mM NaCl, 0.1% Tween-20, pH 8.0. Wells were blocked with 1% BSA in PBS for 2 h at RT to prevent unspecific binding. Serum was diluted to 0.5% in GVB^++^ (5 mM veronal buffer pH 7.4, 144 mM NaCl, 1 mM MgCl_2_, 0.15 mM CaCl_2_, and 1% gelatin) and added to the plates, followed by 1 h incubation at 37°C. Deposited C3b was detected using a goat anti-C3 antibody (ICN pharmaceuticals/Cappel, Aurora, OH, USA) and a rabbit anti-goat HRP conjugate (Dako Denmark A/S, Glostrup, Denmark). Deposited C1q was detected using a biotinylated mouse anti-C1q (clone JL-1) antibody (Hycult Biotech, Uden, The Netherlands) and a streptavidin-HRP conjugate (Thermo Fisher Scientific). The plates were developed using *O*-phenylenediamine substrate (Dako) and H_2_O_2_ and the absorbance at 490 nm was measured.

To study complement activation on CII-bound antibodies, microtiter plates were coated with 10 µg/ml CII in 75 mM sodium carbonate buffer pH 9.6 overnight at 4°C. The wells were blocked using 3% fish gelatin in 50 mM Tris–HCl, 150 mM NaCl, 0.1% Tween-20, pH 8.0 (blocking buffer). M2139 mAb alone at a concentration of 10 µg/ml or in combination with 10, 20, and 40 µg/ml of EndoS-hydrolyzed or unhydrolyzed Hy2.15, L243, or M2139 IgG diluted in blocking buffer was added and the plates were incubated at 4°C overnight. Serum diluted to 4% in DGVB^++^ (2.5 mM veronal buffer pH 7.35, 72 mM NaCl, 0.1% gelatin, 1 mM MgCl_2_, 0.15 mM CaCl_2_, 2.5% glucose) was added to the wells and complement activation was allowed to proceed for 1 h at 37°C. Deposited C3b was detected as above. RF-like activity of CIIC1 antibodies was determined as described earlier (40). Biotin labeled CIIC1 was detected by europium-conjugated streptavidin using the dissociation-enhanced lanthanide fluoroimmunoassay system.

### Analysis of the *In Vitro* Effects of mAbs Using Bovine Cartilage Explants

Articular cartilage samples extracted from adult bovine metacarpophalangeal joints and cartilage shavings (5 mm × 5 mm × 1 mm) were cultured for up to 14 days in DMEM with 20% (v/v) FCS and 25 µg/ml ascorbic acid, having 100 µg/ml of mAb or in medium alone. Medium was changed every 2 day, and fresh ascorbic acid and mAb were added at each change. Cartilage samples were tested in duplicate, and all experiments were performed at least twice. On day 14, cartilage explants were fixed in 4% paraformaldehyde and embedded in paraffin for Fourier transform infrared microspectroscopy (FTIRM). Sections (5 µm) were placed onto MirrIR low-e microscope slides (Kevley Technologies, Chesterland, OH, USA), and adjacent sections were stained with toluidine blue. FTIR images were recorded with a Stingray Digilab FTS 7000 series spectrometer coupled to a UMA 600 microscope equipped with a 64 × 64 focal plane array detector. For each spectrum, 16 scans were co-added at a resolution of 6/cm. The spectra were analyzed using CytoSpec imaging software (41). Raw chemical maps were generated from the integrated intensities of specific functional groups identified in the spectra, and 10 spectra from the surface of the explant and 10 from the interior were extracted from the raw chemical maps. The mean spectra for “surface” and “interior” were calculated to assess the effects of antibody penetration on the peaks characteristic of CII and proteoglycans. Analysis was performed on the location of the amide 1 peak (1,640–1,670/cm), which represents total protein, which for cartilage, is primarily CII. For proteoglycans, analysis was based on the height of the peak at 1,076/cm, within the region of 1,175–960/cm derived from carbohydrate moieties.

### Collagen-Induced Arthritis

Arthritis was induced in male (BALB/c × B10.Q) F1 mice with 100 µg of rat CII/CFA per mouse by i.d. immunization on day 0 and treated i.v. with different test substances on days 14, 21, 28, and 35. Development of clinical arthritis was followed through visual scoring of the animals based on the number of inflamed joints in each paw, starting 2 weeks postimmunization and continuing until the end of the experiment. An extended scoring protocol (25) ranging from 1 to 15 for each paw with a maximum score of 60/mouse was used. The mice were scored three times per week after immunization.

### Statistical Analyses

All the mice with arthritis were included for calculation of severity. The severity of arthritis was analyzed by Mann–Whitney *U* test and the incidence by Chi Square or Fisher exact test using Statview (version 5.0.1) and Prism software. Two-way ANOVA and two-tailed Student’s *t*-test were also used for statistical analysis. Significance was considered when *p* < 0.05, for a 95% confidence interval.

## Results

### Small Amounts of EndoS Inhibit IgG-Mediated Inflammation

**Figure 1:**
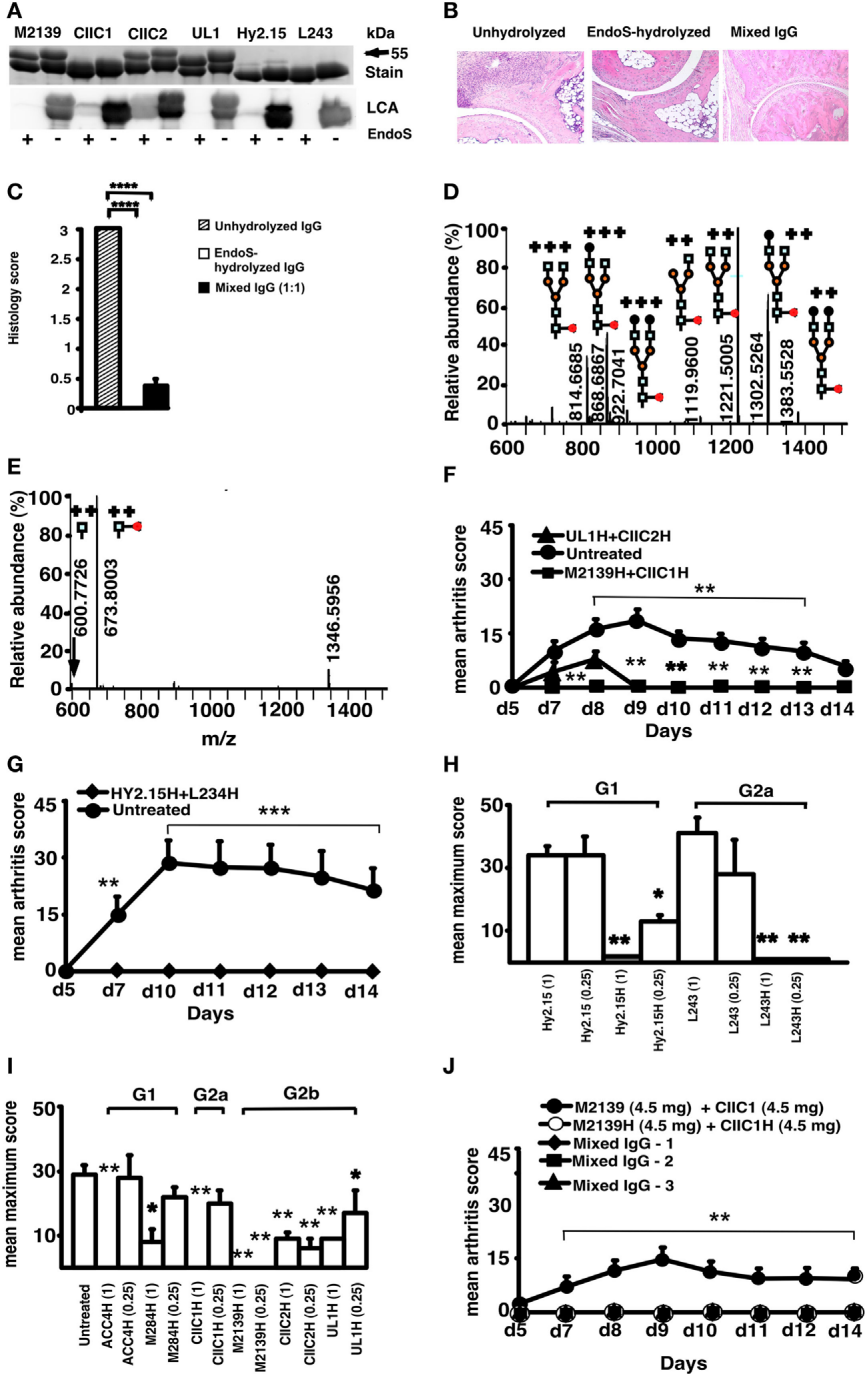
Endo-β-*N*-acetylglucosaminidase (EndoS) and enzyme-hydrolyzed IgG inhibit inflammation. **(A)** SDS/PAGE and lectin blot analysis of mAbs incubated with (+) or without (−) EndoS hydrolysis and separated by 10% SDS/PAGE. The proteins were detected by PageBlue stain (Stain) or by blotting onto a PVDF membrane probed with *Lens culinaris* agglutinin (LCA). **(B)** Representative figures of H&E-stained ankle joints of mice (*n* = 3–4/group) injected with anti-CII mAbs; unhydrolyzed (*Left*), EndoS-hydrolyzed (*Center*), or mixed IgG (*Right*) on day 9. Magnification 10×. **(C)** Joint sections were scored under the microscope based on the scoring scale as described in Section “Materials and Methods.” In each group, at least five sections were scored for each mouse. ****p < 0.0001. Error bars indicate ± SEM. **(D)** Mass spectrometric analysis of Hy2.15 and **(E)** EndoS-treated Hy2.15. Shown spectra were acquired during the time period for which the majority of glycosylated peptides from EEQFNSTFR (21.5–23.0 min) elute. Doubly and triply charged ions as well as predicted glycan structures are shown. All numbers given are for the monoisotopic mass charge. In all of the animal experiments, male (BALB/c × B10.Q) F1 mice were used. Unless otherwise stated all of the mice received antibodies i.v. (d 0) and 25 µg of LPS i.p. (d 5). For arthritis induction in experiments shown in **(F,G,J)**, 9 mg of two anti-CII mAb mixtures (M2139 + CIIC1) were used, whereas for experiments in **(H,I)** 4 mg of four anti-CII mAb mixture (M2139 + CIIC1 + CIIC2 + UL1) was used. Antigen specificity is not required for inhibition. Mice (*n* = 42) were injected with 4 mg of EndoS-hydrolyzed IgG **(F)** M2139H + CIIC1H or UL1H + CIIC2H or **(G)** Hy2.15H + L243H followed by anti-CII mAb. Dose and subclass dependency. **(H)** Mice (*n* = 39) were injected with EndoS-hydrolyzed or unhydrolyzed IgG1 (Hy2.15) or IgG2a (L243) mAb binding to joint unrelated antigens at two different concentrations (1 and 0.25 mg), followed by anti-CII mAb. **(I)** Mice (*n* = 65) were injected with different subclasses of EndoS-hydrolyzed anti-CII (M284H, M2139H, CIIC1H, CIIC2H, and UL1H) or anti-citrullinated CII peptide IgG (ACC4H) at two different concentrations (1 and 0.25 mg), followed by anti-CII mAb. **(J)** Mice (*n* = 25) were injected with a mixture of EndoS-hydrolyzed and/or unhydrolyzed anti-CII IgG at different combinations. In mixed IgG groups, group 1 received 4.5 mg of unhydrolyzed and 4.5 mg of EndoS-hydrolyzed IgG, group 2 had 6.8 mg of unhydrolyzed and 2.3 mg of EndoS-hydrolyzed IgG, and group 3 received 7.9 mg of unhydrolyzed and 1.1 mg of EndoS-hydrolyzed IgG. Error bars indicate ± SEM. Arthritis scoring scale of 0–60 was used (25). All the groups were compared with the untreated group for statistical analysis. *p < 0.05; **p < 0.01; ***p < 0.001.

Endo-β-*N*-acetylglucosaminidase treatment specifically cleaved the Asn-297 glycan on IgG (Figure 1A), which removed almost all (99%) of the variable glycan chains attached to the first *N*-acetylglucosamine (GlcNAc) residue of the Fc region (Figures 1D,E; Tables 1 and 2). Injection of anti-CII mAb (EndoS-unhydrolyzed IgG) induced typical CAIA. Arthritis developed in mice as early as 48 h with 100% incidence at day 10. Massive infiltration of immune cells, pannus formation, and distinct bone and cartilage erosions were observed in this group of mice (Figure 1B). Injection of EndoS-hydrolyzed IgG, irrespective of CII epitope specificity, did not lead to any signs of arthritis (Figures 1B,F–J) resulting in normal joint architecture. Interestingly, mice treated with a mixture of unhydrolyzed and EndoS-hydrolyzed IgG potently blocked the development of arthritis. EndoS-hydrolyzed antibodies against trinitrophenol (TNP) (Hy2.15) and against human HLA-DR (L243), which are commonly used as control antibodies in the mouse, also completely inhibited CAIA when mixed with the anti-CII antibodies used for induction (Figure 1G). Inhibition of arthritis occurred irrespective of the IgG subclass, or the antigen specificity (Figures 1H,I). When we analyzed the dose dependence of the arthritis suppressive effect, we found that 1 mg of EndoS-hydrolyzed IgG present in the mixture of 9 mg of antibodies completely inhibited arthritis (Figure 1J), whereas 3 mg of unmodified anti-CII mAb was sufficient to induce arthritis (20).

**Table 1:** IgG glycoforms before and after endo-β-*N*-acetylglucosaminidase (EndoS) hydrolyzation.

Antibody		M2139	M2139H	CIIC1	CIIC1H
Sequence		EDYNSTIR	EDYNSTIR	EDYNSTLR	EDYNSTLR
Glycan^a^	HexNAc(1)	–	2.6%	–	3.8%
	HexNAc(1)dHex(1)	–	97%	–	95%
	HexNAc(4)Hex(3)	–	–	–	–
	HexNAc(4)Hex(4)	–	–	–	–
	HexNAc(3)Hex(3)dHex(1)	1.5%	–	6.5%	–
	HexNAc(3)Hex(4)dHex(1)	0.4%	–	1.3%	–
	HexNAc(4)Hex(3)dHex(1)	48%	–	35%	0.7%
	HexNAc(4)Hex(4)dHex(1)	43%	–	42%	0.5%
	HexNAc(4)Hex(5)dHex(1)	4.3%	–	4.4%	–
	HexNAc(4)Hex(6)dHex(1)	0.3%	–	0.2%	–
	HexNAc(3)Hex(4)dHex(1)NeuGc(1)	–	–	5.0%	–
	HexNAc(4)Hex(4)dHex(1)NeuGc(1)	0.5%	–	3.2%	–
	HexNAc(4)Hex(5)dHex(1)NeuGc(1)	1.8%	–	1.8%	–
	HexNAc(4)Hex(6)dHex(1)NeuGc(1)	–	–	0.3%	–

*^a^HexNAc, N-acetylhexoseamine; dHex, deoxy-hexose; Hex, hexose; NeuGc, N-glycolylneuraminic acid; H, EndoS-hydrolyzed*.

*Relative abundances (%) of the main Fc IgG glycoforms found in the antibodies and EndoS treated antibodies, respectively. Glycopeptides were identified by their characteristic retention times and accurate monoisotopic masses (within <10 ppm from the theoretical values) of doubly and triply charged ions. Peptide sequences and glycan compositions are indicated. Glycans substituting EDY**N**STIR and EDY**N**STLR eluted at approximately 18–20 min and EEQF**N**STFR at approximately 21–23 min, respectively*.

**Table 2:** Joint unrelated antigen(s)-specific IgG glycoforms before and after endo-β-*N*-acetylglucosaminidase (EndoS) hydrolyzation.

Antibody		L243	L243H	Hy2.15	Hy2.15H
Sequence		EDYNSTLR	EDYNSTLR	EEQFNSTFR	EEQFNSTFR
Glycan^a^	HexNAc(1)	–	2.6%	–	15%
	HexNAc(1)dHex(1)	–	97%	–	84%
	HexNAc(4)Hex(3)	–	–	3.4%	–
	HexNAc(4)Hex(4)	–	–	1.3%	–
	HexNAc(3)Hex(3)dHex(1)	4.6%	–	2.5%	–
	HexNAc(3)Hex(4)dHex(1)	3.3%	–	0.7%	–
	HexNAc(4)Hex(3)dHex(1)	22%	–	46%	0.1%
	HexNAc(4)Hex(4)dHex(1)	50%	–	27%	0.5%
	HexNAc(4)Hex(5)dHex(1)	10%	–	5.0%	0.2%
	HexNAc(4)Hex(6)dHex(1)	4.6%	–	0.1%	–
	HexNAc(3)Hex(4)dHex(1)NeuGc(1)	2.6%	–	2.5%	–
	HexNAc(4)Hex(4)dHex(1)NeuGc(1)	0.3%	–	5.9%	–
	HexNAc(4)Hex(5)dHex(1)NeuGc(1)	1.7%	–	4.5%	–
	HexNAc(4)Hex(6)dHex(1)NeuGc(1)	0.7%	–	1.5%	–

*^a^HexNAc, N-acetylhexoseamine; dHex, deoxy-hexose; Hex, hexose; NeuGc, N-glycolylneuraminic acid; H, EndoS-hydrolyzed*.

*Relative abundances (%) of the main Fc IgG glycoforms found in the antibodies and EndoS treated antibodies, respectively. Glycopeptides were identified by their characteristic retention times and accurate monoisotopic masses (within <10 ppm from the theoretical values) of doubly and triply charged ions. Peptide sequences and glycan compositions are indicated. Glycans substituting EDY**N**STIR and EDY**N**STLR eluted at approximately 18–20 min and EEQF**N**STFR at approximately 21–23 min, respectively*.

**Figure 2:**
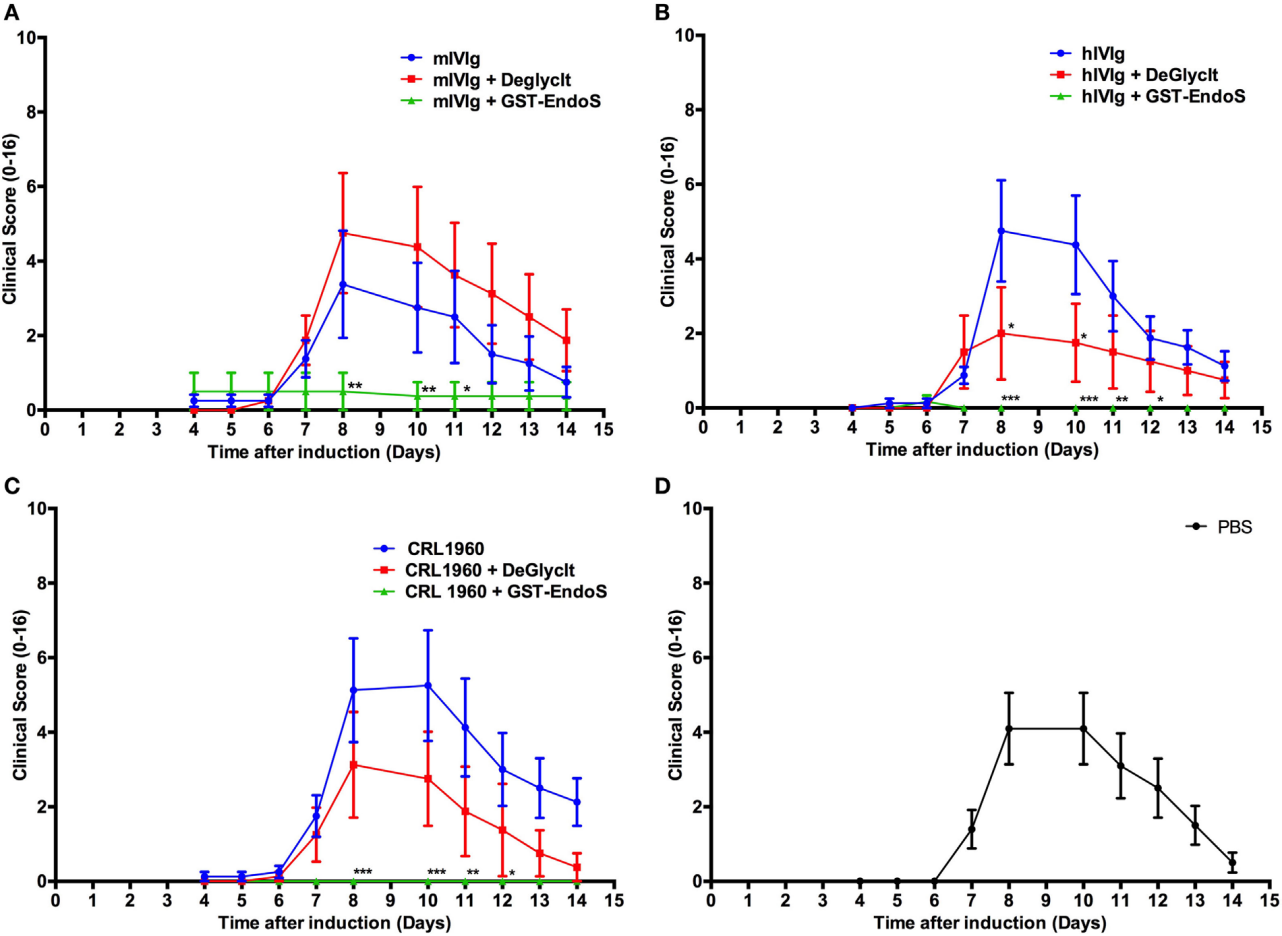
Analysis of endo-β-*N*-acetylglucosaminidase (EndoS) effects *in vivo*. Arthritic score (mean ± SEM) of collagen antibody-induced arthritis animals (*n* = 8–10 mice/group) treated with different antibody preparations that were either untreated or deglycosylated with GST-tagged EndoS (GST-EndoS) or EndoS immobilized to sepharose beads (DeGlycIt). Arthritis was induced on day 0 by i.p. administration of 2 mg/mouse of anti-collagen antibody cocktail followed by administration of 50 µg LPS on day 5. The test substances (1 mg/mouse) were injected i.v. on day 0. DeGlycIT or GST-EndoS treated mouse intravenous immunoglobulins (mIVIg) **(A)**, human intravenous immunoglobulins (hIVIg) **(B)** or mouse monoclonal antibody recognizing trophozoite antigens (2B5.3; CRL-1960) **(C)** were used as test substances and PBS was injected as control **(D)**. Error bars indicate ± SEM. Arthritis scoring scale (0–16) was used (25). All the groups were compared with the untreated group for statistical analysis. *p < 0.05; **p < 0.01; ***p < 0.001.

We have earlier reported our results interpreted as dominant suppression of inflammation by glycan hydrolyzed IgG (42) but we withdrew this report as we discovered that we could not rule out that small amounts of EndoS, undetectable on PageBlue stained gels, remained after hydrolysis *in vitro*. Consequently, when we used DeglycIT column for cleaving the N-linked sugars instead of GST-EndoS treatment and serial passage through glutathione-Sepharose 4B columns (DeglycIT spin column contains EndoS immobilized on agarose, which facilitates complete removal of the enzyme in one step instead of two step process with GST-EndoS treatment and purification using glutathione-Sepharose 4B), we observed no inhibition of arthritis (Figures 2A–C) compared with the control mice (Figure 2D). We rechecked our GST-EndoS treated purified IgG samples used in the above experiments by Western blot and in most (12/14) of the samples we could still not detect the presence of EndoS. In the remaining two samples, we found very low level of residual enzyme, <0.02% of total protein (data not shown).

**Figure 3:**
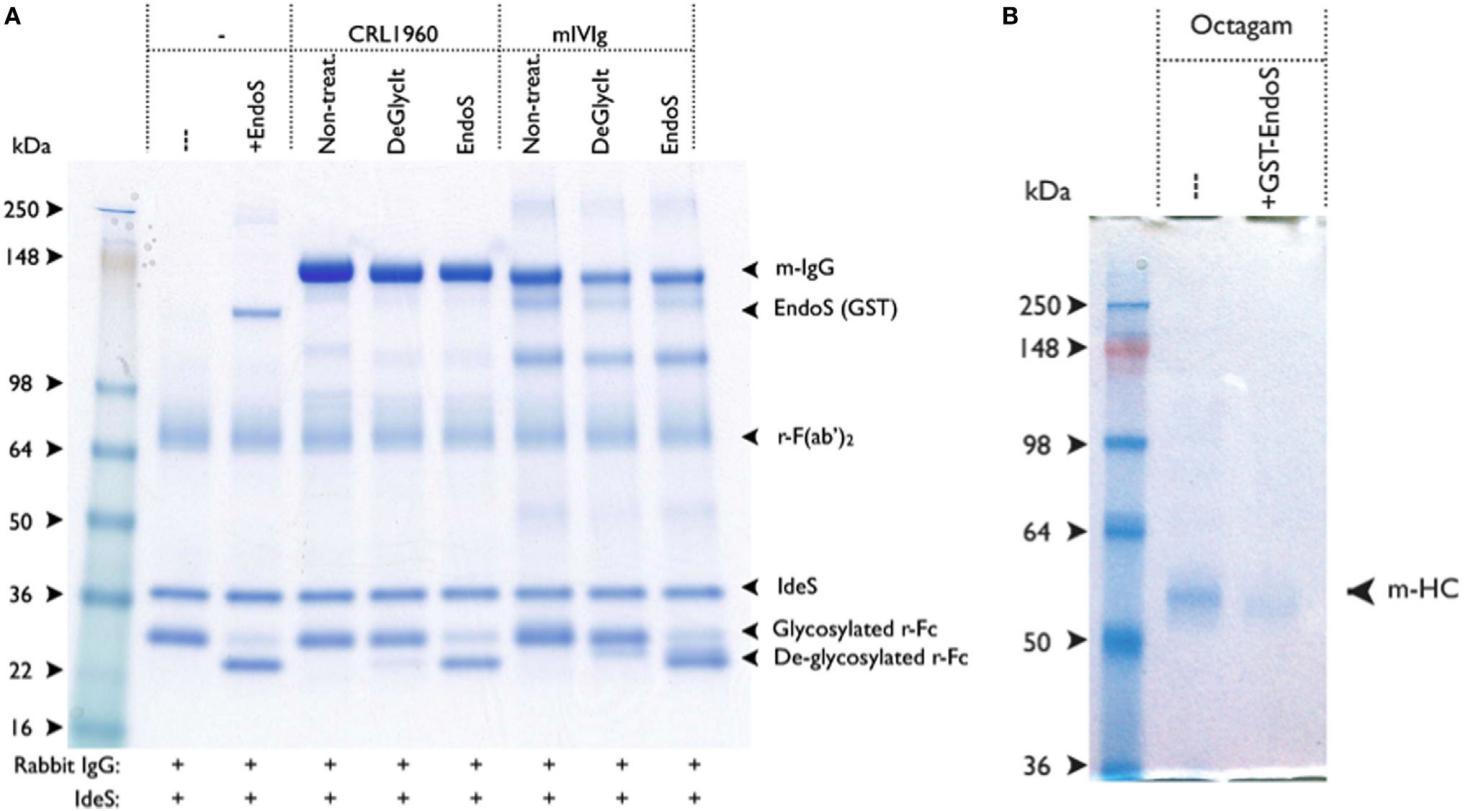
IgG treated with GST-endo-β-*N*-acetylglucosaminidase (EndoS) contains residual enzyme activity. **(A)** EndoS treated antibody preparations were tested for residual EndoS activity by using rabbit IgG cleavage assay. Rabbit IgG was used as a reporter for EndoS activity, in which ability of IdeS to efficiently cleave the heavy chain of rabbit IgG was used. After IdeS treatment, the deglycosylated rabbit Fc-fragment was distinguished from the non-deglycosylated rabbit Fc-fragment using SDS-PAGE (i.e., a 25-kDa fragment ± glycosylation). Rabbit IgG (1.7 µg) and IdeS (0.3 µg) were added to each of the sample. Molecular weight markers (lane 1); without EndoS (lane 2); with EndoS (0.5 µg, lane 3); 1.7 µg of CRL-1940 untreated (lane 4); CRL-1940 DeGlycIT treated (lane 5); CRL-1940 GST-EndoS treated (lane 6); mouse intravenous immunoglobulins (mIVIg) untreated (lane 7); mIVIg DeGlycIT treated (lane 8); mIVIg GST-EndoS treated (lane 9). m-IgG: mouse IgG, r-Fc: monomeric rabbit Fc-fragment. The gel shown was run under non-reducing condition. **(B)** BALB/c mice were intravenously injected with untreated control Octagam (human intravenous immunoglobulins, 1 mg) or GST-EndoS treated Octagam (1 mg). Serum was collected 18 h after injection and IgG was purified from serum using protein G sepharose. Samples were treated with IdeS to cleave remaining Octagam and the samples were analyzed on SDS-PAGE. m-HC, mouse heavy chain. IgG purified from mice injected with untreated control Octagam (lane 1) or GST-EndoS treated Octagam (lane 2) were shown. The gel shown was run under reducing condition.

To find whether the residual EndoS present in the IgG preparation had any enzymatic activity, we designed a new rabbit IgG cleavage assay using another streptococcal enzyme, IdeS. With this assay, it is now possible to detect the presence of residual activity of EndoS in the IgG preparation treated with EndoS even after three serial passages over Glutathione-Sepharose 4B columns with a 1,000-fold overcapacity of GST binding but not after treatment with DeglycIT column (Figure 3A).

**Figure 4:**
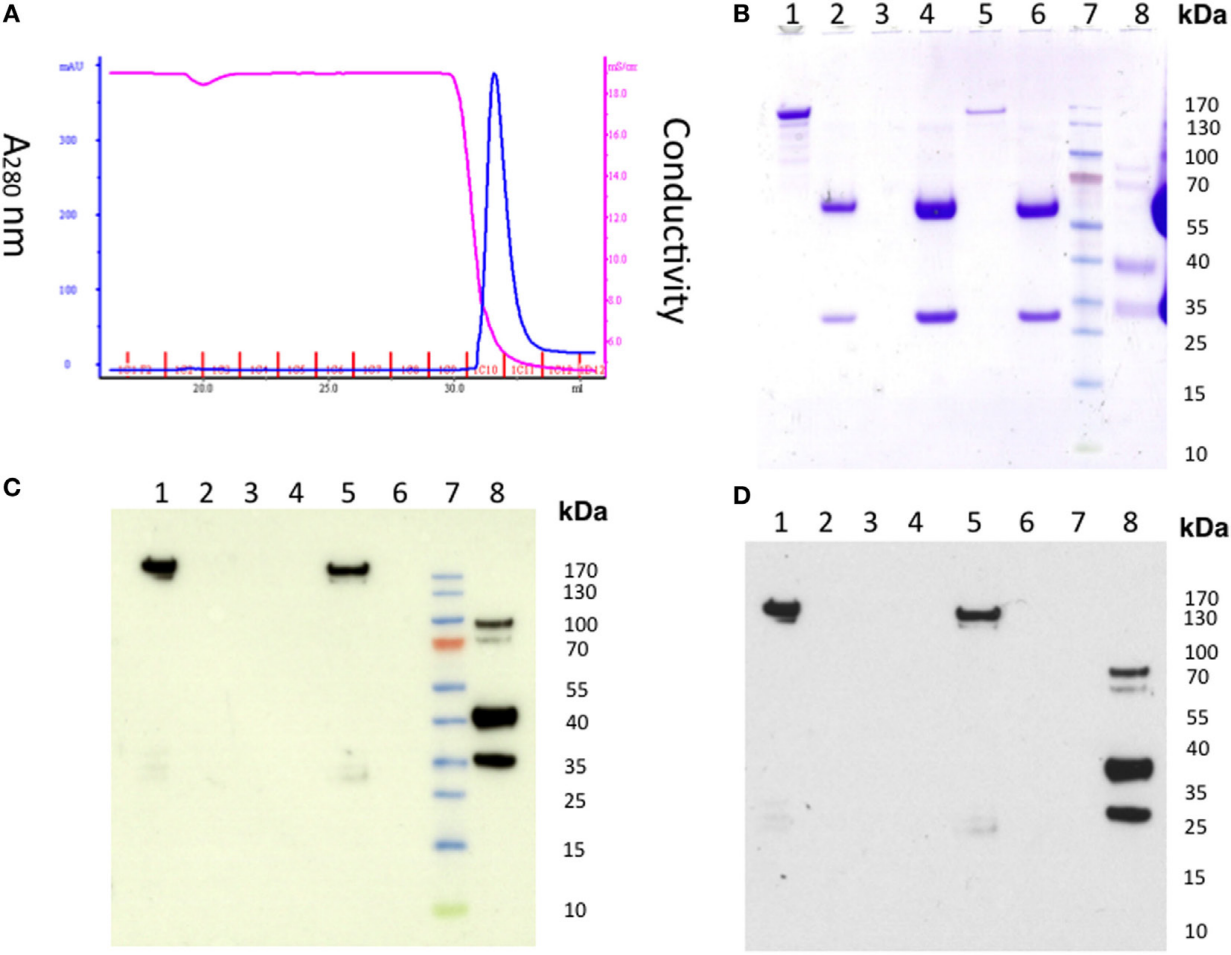
Detection of residual endo-β-*N*-acetylglucosaminidase (EndoS) in the GST-EndoS treated IgG after protein G purification. M2139 antibody (2 mg) was incubated with 0.1 µg or 1 µg of EndoS in PBS at 37°C for 1 h followed by immediate purification on HiTrap Protein G HP column **(A)** Chromatogram. The flow through fraction and the eluate were analyzed by SDS-PAGE followed by Western blot. **(B)** SDS-PAGE and Western blot using **(C)** alkaline phosphatase or **(D)** peroxidase conjugate as secondary antibody. Flow-through samples were concentrated before loading on to SDS-PAGE. After transfer, the Western blot membrane was incubated with Eu-N1-labeled anti-GST antibody, followed by donkey anti-goat AP or anti-goat HRP conjugate. For the AP conjugate, the positive bands were visualized using 1-Step TM NBT/BCIP substrate. For the HRP conjugate, the light emission was enhanced using ECL followed by film exposure. Lane 1, EndoS; lane 2, M2139; lane 3, concentrated flow through (0.1 mg group); lane 4, eluate (0.1 mg group); lane 5, concentrated flow through (1 mg group), lane 6, eluate (1 mg group); lane 7, protein standard; lane 8, control GST-fusion protein.

To further investigate if the level of residual EndoS activity was sufficient to deglycosylate the mouse endogenous IgG pool *in vivo*, mice were injected with 1 mg of untreated hIVIg (Octagam) or GST-EndoS treated Octagam. IgG was purified from the mouse serum (collected 18 h later) using protein G Sepharose and the samples were treated with IdeS to cleave the human IgG. The results indicate that the intact (i.e., of mouse origin) IgG heavy chain band migrates a longer distance (indicating a decreased molecular size) in the sample purified from the mouse injected with the GST-EndoS treated Octagam compared with the heavy chain band from the mouse injected with the untreated control Octagam (Figure 3B). Hence, de-glycosylation is a likely explanation for this shift in size on SDS-PAGE. As a next step, we titrated the amount (100 µg to 1 ng) of EndoS needed for inhibiting antibody-mediated inflammation (Table 3). Our results show that intravenous injection of as low as 1 µg EndoS was sufficient to completely inhibit antibody-initiated inflammation and even lower when mixed with DeglycIT treated anti-CII antibody, 0.1 µg EndoS (Table 3). As a next step, we incubated antibodies with recombinant EndoS at two different concentrations (0.1 or 1 µg) and after incubation for 1 h antibodies were purified using affinity column or left unpurified (Figure 4). These antibodies were injected into mice before arthritogenic monoclonal antibodies were transferred into them. Interestingly, as observed earlier, 1 µg but not 0.1 µg of EndoS added to the purified DeglycIT treated antibody was sufficient to inhibit arthritis, whether it underwent one more cycle of affinity purification after incubation with EndoS or not (Table 3). However, deglycIT purified M2139 added with 1 µg of GST-EndoS was found to be inferior to GST-EndoS hydrolyzed M2139 in attenuating joint inflammation (Table 3).

**Table 3:** Titration of inhibitory dose of endo-β-*N*-acetylglucosaminidase (EndoS) using antibody-mediated inflammation.

Groups^a^	Incidence	Maximum arthritis score^b^ (mean ± SEM)
PBS	16/18	21 ± 7
GST-EndoS (100 µg)	0/4**	0**
GST-EndoS (10 µg)	0/4**	0**
GST-EndoS (1 µg)	0/13****	0****
GST-EndoS (0.1 µg)	13/15^n.s.^	18 ± 9^n.s.^
M2139-E^c^	0/4**	0**
M2139-D^d^	14/14^n.s.^	18 ± 6^n.s.^
M2139-D + GST-EndoS (1 µg)^e^	4/9*	10 ± 6*
M2139-D + GST-EndoS (0.1 µg)^e^	9/13^n.s.^	23 ± 4^n.s.^
M2139-D + GST-EndoS (1 µg)^f^	6/9^n.s.^	12 ± 6*
M2139-D + GST-EndoS (0.1 µg)^f^	8/9^n.s.^	21 ± 5^n.s.^

*^a^All the mice received 9 mg of arthritogenic two mAb cocktail (M2139, CIIC1) i.v. on day 0 and 25 µg of LPS was injected i.p. on day 5. Four-month-old male (BALB/c × B10.Q) F1 mice (*n* = 112) were used in all these experiments*.

*^b^Arthritis severity was scored using the scale 0–60. Mice with arthritis only were included for calculations*.

*^c^M2139 antibodies (250 µg) treated with GST-EndoS was injected i.v. on day 0*.

*^d^M2139 antibodies (250 µg) treated with DeglycIT and purified was injected i.v. on day 0*.

*^e^M2139 antibodies (250 µg) treated with DeglycIT and purified were incubated with indicated concentrations of GST-EndoS for 1 h at RT before i.v. injection on day 0*.

*^f^M2139 antibodies (250 µg) treated with DeglycIT and purified were incubated with indicated concentrations of GST-EndoS for 1 h at RT. Antibodies were further purified using HiTrap Protein G HP column before i.v. injection on day 0*.

*p < 0.05; **p < 0.01; ****p < 0.0001; n.s., not significant.

**Figure 5:**
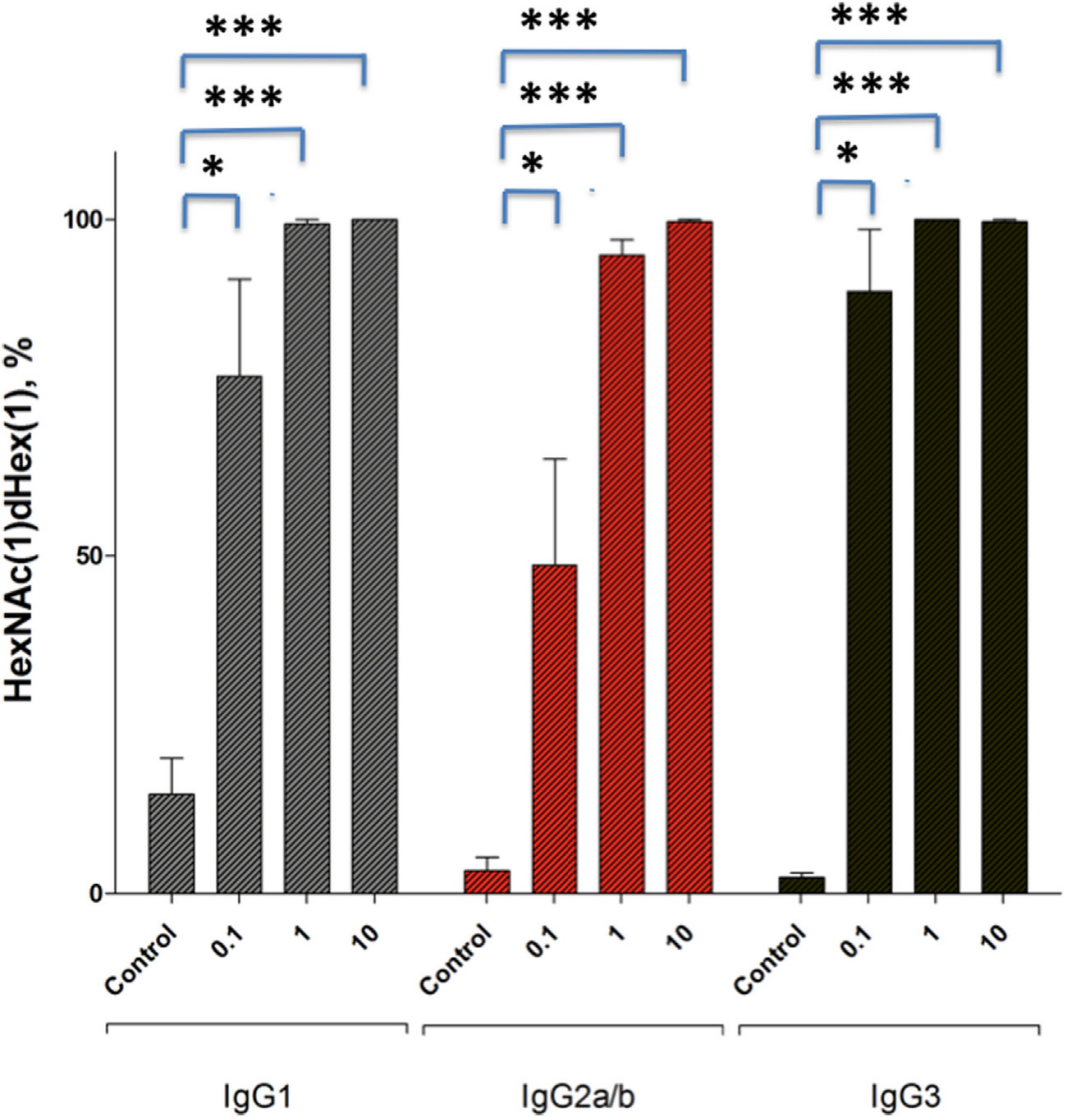
Relative abundance of dHex-HexNAc after endo-β-*N*-acetylglucosaminidase (EndoS) treatment *in vivo*. Male (BALB/c × B10.Q) F1 mice (*n* = 12) were injected with GST- recombinant protein (control), 0.1 µg, 1 µg, or 10 µg of GST-EndoS. Serum samples were collected before and 18 h after the injection. Even at 0.1 µg of EndoS injection the majority of the found glycopeptides, for all IgG types are the truncated dHex-HexNAc variants. The bar graph shows mean ± SEM of the relative abundance of dHex-HexNAc compared with other Fc-glycopeptides. dHex(1)-HexNAc (1), dHex (1)Hex (3)HexNac (4), dHex (1)Hex (4)HexNAc (4), dHex (1)Hex (5)HexNAc (4) sugars present on IgG1 (EEQFNSTFR), IgG2b (EDYNSTIR)/IgG2a (EDYNSTLR), and IgG3 (EAQYNSTFR) peptides, respectively. Glycopeptides were analyzed using mass spectrometry. *p < 0.05; ****p < 0.0005.

From these results, we concluded that the small amount of residual EndoS present in enzyme treated IgG fractions is a requirement for the observed hydolyzed antibody-mediated arthritis inhibition. To determine whether this small amount of EndoS was sufficient to cleave the sugars from IgG *in vivo* we injected small amounts of EndoS and analyzed the presence of glycan on circulating IgG. As shown in Figure 5, it is clear that 0.1 µg of EndoS injected *in vivo* was capable of specifically cleaving most of the carbohydrates present on all the IgG subclasses, whereas only at 1 µg concentration all the carbohydrates present in the Fc region of IgG were cleaved.

**Figure 6:**
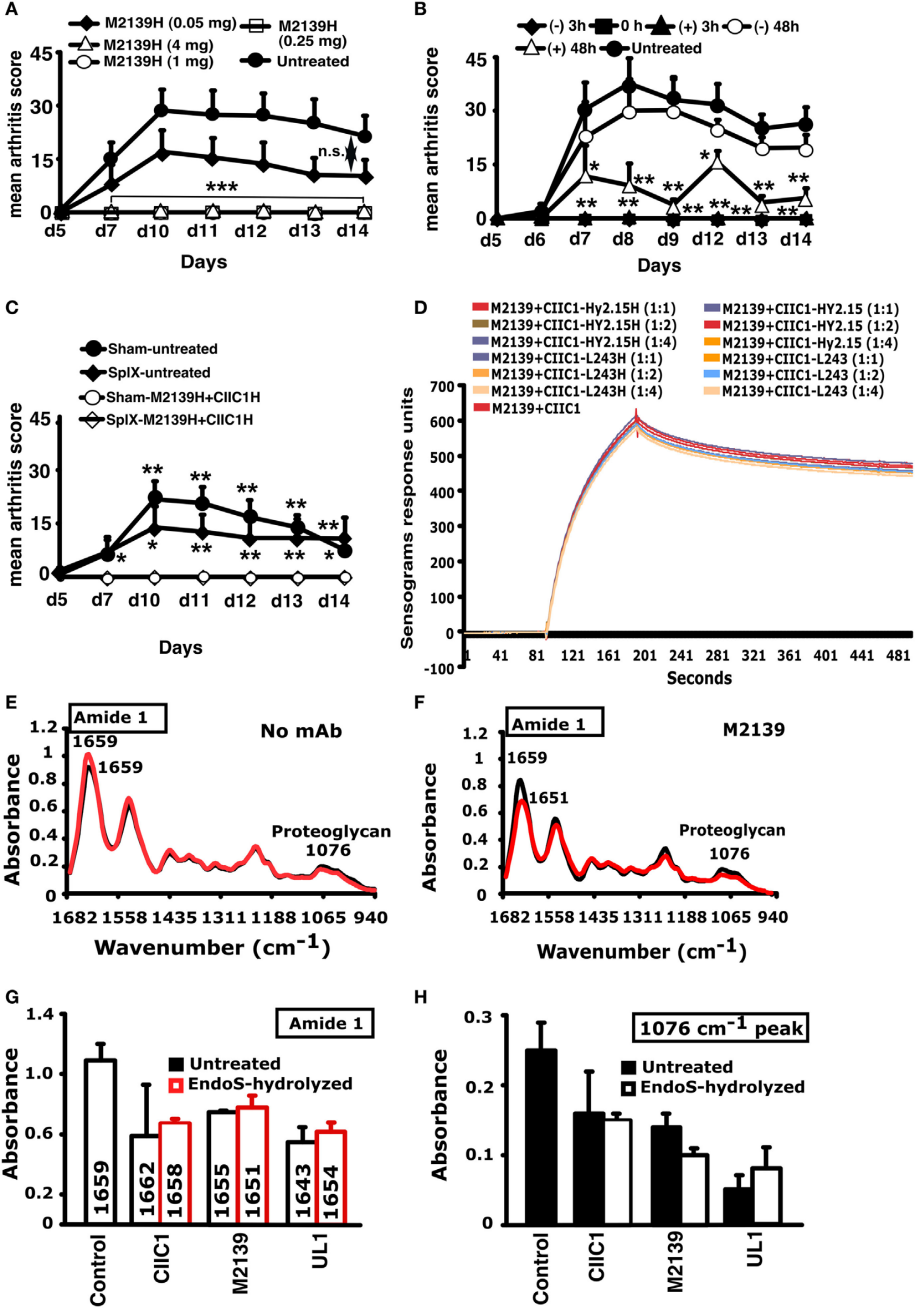
Inhibition of inflammation and Surface Plasmon Resonance (SPR) and Fourier transform infrared microspectroscopy (FTIRM) analysis. **(A)** Mice (*n* = 25) were injected with different concentrations (50–4,000 µg) of endo-β-*N*-acetylglucosaminidase (EndoS)-hydrolyzed single anti-CII IgG (M2139H), followed by anti-CII mAb. Three hours after the antibody transfer, LPS was injected. H denotes EndoS-hydrolyzed IgG. Hy2.15 and L243 represent mAbs binding to TNP hapten and human HLA-DR antigen, respectively. **(B)** Mice (*n* = 30) were injected with 1 mg of EndoS-hydrolyzed anti-CII IgG (M2139H + CIIC1H + CIIC2H + UL1H) at different time points (−48, −3, 0, +3, or +48 h). At 0 and 3 h, anti-CII mAb (M2139 + CIIC1 + CIIC2 + UL1) and then LPS were injected. One group of mice received no treatment. **(C)** Effect of splenectomy. Mice (*n* = 21) were either splenectomized (Splx) or sham-operated (Sham). Three weeks later, they were injected with 4 mg of EndoS-hydrolyzed IgG (M2139H + CIICH) or left untreated, followed by anti-CII mAb (M2139 + CIIC1). Panel **(H)** denotes EndoS-hydrolyzed IgG. Error bars indicate ± SEM. **(D)** SPR (Biacore) analysis of antibody binding capacity of EndoS-hydrolyzed and unhydrolyzed IgG was performed using CII immobilized on CM5 sensor chip. MAbs were injected at different concentrations through flow cells at a flow rate of 30 µl/min. Antibodies were injected for 3 min and dissociation of bound molecules was observed for 7 min. There was no difference in antibody binding when EndoS-hydrolyzed or unhydrolyzed IgGs were added at different ratios to anti-CII mAb mixture. **(E,F)** Changes in the chemical composition of the cartilage were assessed using FTIRM analysis. Representative mean spectra are shown from cartilage cultures without antibody **(E)**, and from cartilage cultured for 14 days with 100 µg/ml of unhydrolyzed mAb M2139 **(F)**. The results shown are the mean of 10 measurements taken from the central areas (red line) and near the surface of the tissue (black line). The mean spectra for surface and interior were calculated to assess the effects of antibody penetration on the peaks characteristic of CII and of proteoglycans. **(G,H)** The mean peaks from the surface cartilage were compared with those from antibody-exposed surface of cartilage exposed to the EndoS-hydrolyzed or unhydrolyzed IgG. Cartilage exposed to either EndoS-hydrolyzed or unhydrolyzed IgG (CIIC1, M2139, and UL1) showed similar changes. **(G)** The height and location of the amide 1 peak, which represents the total protein content of the tissue, in the region 1,600–1,700/cm. **(H)** The height of the peak at 1,076/cm represents proteoglycans. All the groups were compared with the untreated group for statistical analysis. *p < 0.05; **p < 0.01; ***p < 0.001; n.s., not significant.

To find the effective therapeutic window, EndoS-hydrolyzed IgG was administered to groups of mice at different concentrations and time points and one group of mice was left untreated in each experiment. Dose titration of the EndoS-hydrolyzed IgG separately showed that complete inhibition of arthritis was achieved down to 250 µg (Figure 6A). In the time titration experiments, antibody cocktail and then LPS were injected at 0 and 3 h, respectively. In the 0 h treatment group, EndoS-hydrolyzed IgG was injected initially, followed by an injection of mAb cocktail. Unlike −48 h time, complete blocking of arthritis was observed when the treatment with EndoS-hydrolyzed IgG was done 3 h before or after the arthritogenic cocktail injection (Figure 6B). However, when EndoS-hydrolyzed IgG was injected 48 h after mAb injection (+48 h group), significant blocking of arthritis was observed (*p* < 0.05 to *p* < 0.01 for different time points), which increases the therapeutic value of the EndoS. Since anti-CII antibodies are bound to cartilage within 30 min after injection as detected by immunohistochemistry (43), we concluded that EndoS and the enzyme hydrolyzed IgG could block the disease most effectively if injected within 48 h after antibody binding or during the time when antibodies start binding to the cartilage surface. However, we did not find any role for the spleen (mice splenectomized or sham operated 2 weeks earlier were used for this purpose) in EndoS and enzyme-hydrolyzed IgG induced arthritis inhibition (Figure 6C).

### EndoS Hydrolysis of IgG Does Not Affect Antigen Binding

Biacore analysis of the antigen-antibody binding in the presence or absence of EndoS and enzyme-hydrolyzed IgG clearly demonstrated that removal of carbohydrate moieties from mAbs did not affect their high affinity binding to CII epitopes (Figure 6D). We have earlier shown antibody-mediated damage in cartilage explants cultured *in vitro* with the mAb to CII used in this study: this pre-inflammatory effect does not require live cells, is mediated by F_ab_ and is epitope dependent (44). To test whether EndoS hydrolysis could change this effect, FTIRM was used for chemical analysis of cartilage (41) cultured in the presence of 100 µg/ml EndoS-hydrolyzed IgG containing minor amounts of the enzyme or unhydrolyzed mAb (Figures 6E–H). The height and location of the amide 1 peak, representing protein, predominantly collagen, and the height of the proteoglycan peak at 1,076/cm were examined at the cartilage surface, where the mAb penetrates, and in the interior of cartilage explants (Figure 6E). After 14 days, the amide 1 peak at the surface and in the interior of the control cartilage cultured without mAb was at 1,659/cm (range 1,655–1,666/cm). There were striking changes in spectra from cartilage cultured in the presence of unhydrolyzed M2139 mAb (Figure 6F). However, the spectra [amide 1 peak representing mainly collagen (Figure 6G) and proteoglycan peak at 1,076/cm (Figure 6H)] obtained were similar when the cartilage was cultured with Endo-S hydrolyzed IgG containing minor amounts of the enzyme or unhydrolyzed anti-CII IgG. Beyond the region of penetration by mAb, the spectra were generally similar to those of controls cultured without mAb, but spectra from the surface of the cartilage showed substantial changes (Figures 6E,F). For both unhydrolyzed and EndoS-hydrolyzed anti-CII containing minor amounts of the enzyme, there was a shift in the location of the amide 1 peak from 1,659/cm to as low as 1,643/cm (Figure 6G). This shift is indicative of denaturation of the CII and accompanied by substantial decreases in the height of the amide 1 peak, as well as the proteoglycan peak at 1,076/cm, indicating a total loss of matrix (Figure 6H). These data confirm that the EndoS-hydrolyzed IgG retained its antibody reactivity.

### EndoS and Enzyme Hydrolyzed IgG Disrupt Larger IC Formation

**Figure 7:**
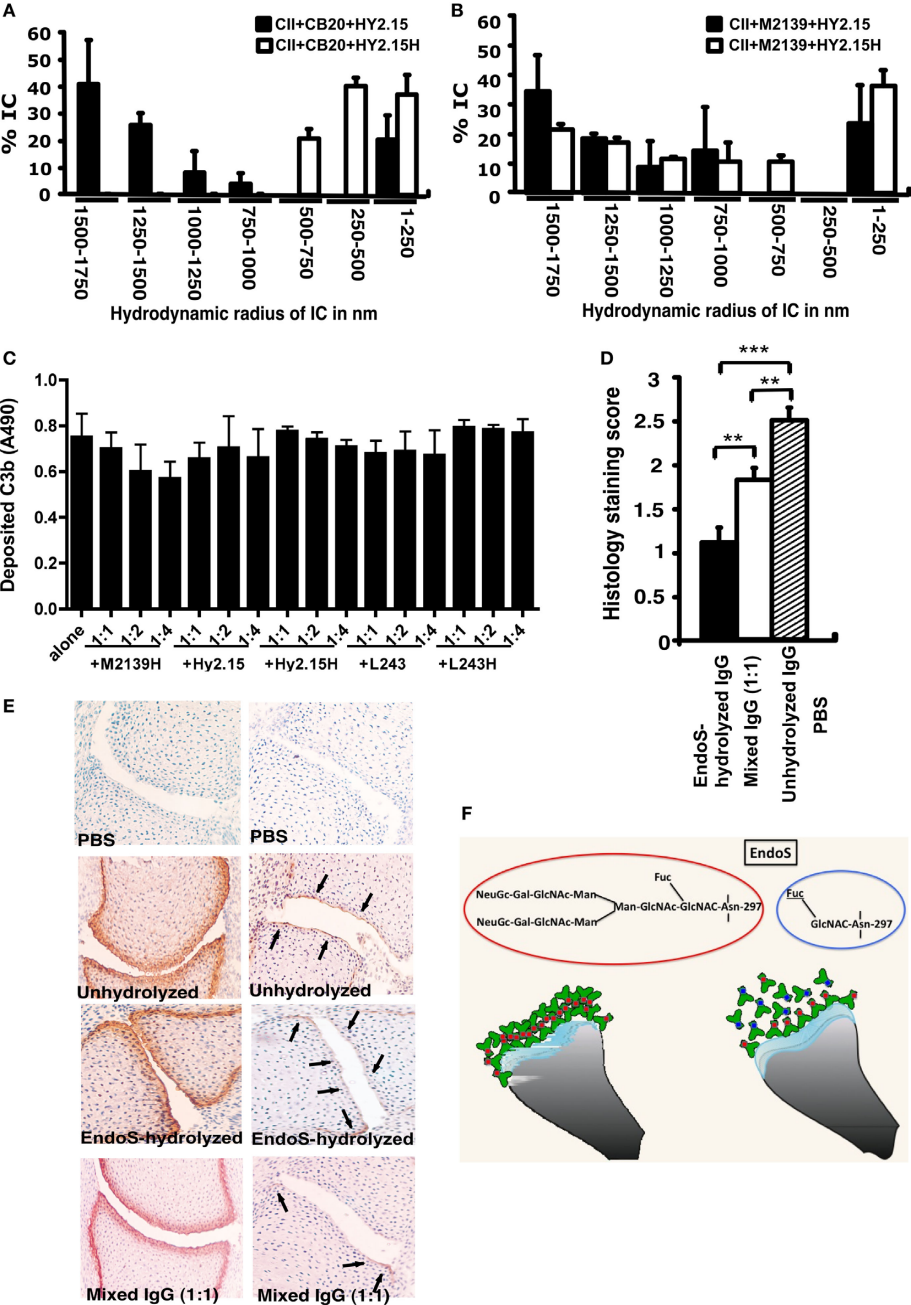
Disturbance of stable ICs and complement activation. Collagen type II (CII; 1 mg/ml) and anti-CII mAb CB20 [low affinity; **(A)**] or M2139 [high affinity; **(B)**] at 1 mg/ml were mixed together at 1:1 ratio and incubated for 30 min at 37°C, followed by addition of anti-hapten IgG, either unhydrolyzed (Hy2.15) or endo-β-*N*-acetylglucosaminidase (EndoS)-hydrolyzed IgG (Hy2.15H) containing minor amounts of EndoS at the ratio 1:1:1. Twenty microliters of this mixture were loaded on to capillary tube in the Dynamic Light Scattering instrument. Relative sizes of ICs present in the solution are indicated in the X-axis and Y-axis denotes percentage of ICs present in the solution. Each sample was measured 5–7 times and the bars represent mean values from two experiments. Error bars indicate ± SEM. **(C)** Complement activation on CII bound anti-CII antibodies (M2139H) were monitored by measuring C3b deposition. Each bar represents mean values from three experiments ± SD. **(D)** Deposition of C3b on the cartilage of mice was used as a measure of IC deposition and complement activation after the injection of EndoS-hydrolyzed containing minor amounts of EndoS, unhydrolyzed or mixed anti-CII IgG. Two- to three-day-old mouse pups (3–4 mice/group) were injected with 1 mg each of unhydrolyzed IgG (M2139 + CIIC2 + UL1), EndoS-hydrolyzed IgG (M2139H + CIIC2H + UL1H) containing minor amounts of EndoS or a mixture of IgGs at 1:1 ratio. In each group, 26–44 joints were scored in total. **p < 0.01; ***p < 0.005. Error bars indicate ± SEM. **(E)** Paw samples collected 24 h later were stained with biotinylated anti-kappa (left column) or goat anti-mouse anti-C3c antibodies (right column). Joint sections from PBS (first row), unhydrolyzed (second row), EndoS-hydrolyzed containing minor amounts of EndoS (third row), or a mixture (1:1) of IgG (fourth row) injected mice is shown. Magnification 20×. Arrows indicate C3b deposition within ICs formed on the joint cartilage surface. **(F)** Diagram illustrating possible binding mechanisms involved in the suppression of arthritis by EndoS-hydrolyzed antibodies containing minor amounts of EndoS.

As a next step, we analyzed whether the EndoS and EndoS-hydrolyzed antibodies can disrupt the growth of Fc-dependent ICs. For this we used DLS technique and analyzed the formation of IC by CII and anti-CII mAb in the presence of EndoS-hydrolyzed IgG, which contain minor amounts of the enzyme or unhydrolyzed IgG. As shown in Figures 7A,B, larger IC formation was clearly disturbed by the presence of EndoS and enzyme-hydrolyzed IgG. Interestingly, disturbance of large IC was more prominent in the presence of low affinity (Figure 7A) than high affinity IgG (Figure 7B). It has earlier been shown that the binding to low affinity Fc-receptors is decreased by EndoS modification (21). However, the binding is directly related to IC formation. Different data have been reported regarding complement binding after EndoS hydrolysis (21, 22). To directly investigate this, we analyzed complement fixation using either immobilized EndoS-hydrolyzed IgG containing residual amount of EndoS or unhydrolyzed IgG, or CII-anti-CII ICs. The presence of the enzyme and EndoS-hydrolyzed IgG did not interfere with the ability of antibodies to stimulate complement deposition, when bound to immobilized CII (Figure 7C). Furthermore, presence of the enzyme and EndoS-hydrolyzed IgG did not interfere with the ability of antibodies to stimulate complement deposition, when antibodies were immobilized directly on the plate (data not shown).

The presence of EndoS-hydrolyzed IgG did not affect complement activation but did affect IC stability *in vitro*; hence, we further analyzed deposition of C3b, the activated product of complement factor C3, on the cartilage of mice as a measure of IC deposition and complement activation *in vivo*. Twenty-four hours after the injection of EndoS-hydrolyzed, unhydrolyzed or mixed anti-CII IgG, mouse paws were analyzed for the binding of mAbs to cartilage using anti-kappa antibodies as well as for deposited C3b. The mAbs readily bound to the cartilage surface, but the pattern of C3b deposition between the groups was entirely different (Figures 7D,E). Minimal C3b staining was observed, only on the sub-chondral bone junction area of the joints from mice injected with EndoS-hydrolyzed IgG compared with a significant level of deposition throughout the cartilage surface in the unhydrolyzed IgG injected group. In mice injected with mixed IgG, staining on the sub-chondral bone junction area was more intense with weak staining on the cartilage surface.

**Figure 8:**
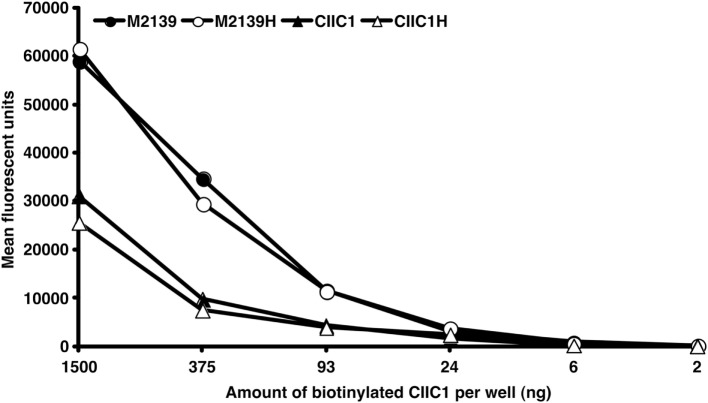
Disturbance of IC formation by rheumatoid factor (RF)-like activity of CIIC1 mAb. Endo-β-*N*-acetylglucosaminidase (EndoS) hydrolyzed (M2139H, CIIC1H) or unhydrolyzed (M2139, CIIC1) antibody coated (10 µg/well) ELISA plates after blocking with BSA were incubated with different concentrations of biotinylated CIIC1 antibody. Europium-conjugated streptavidin and the dissociation-enhanced lanthanide fluoroimmunoassay system were used for detection of biotinylated antibody. There was no significant difference in biotinylated CIIC1 antibody binding to EndoS hydrolyzed or unhydrolyzed M2139 and CIIC1 mAbs demonstrating negligible contribution of RF like activity of CIIC1 mAb in disturbing IC formation.

**Figure 9:**
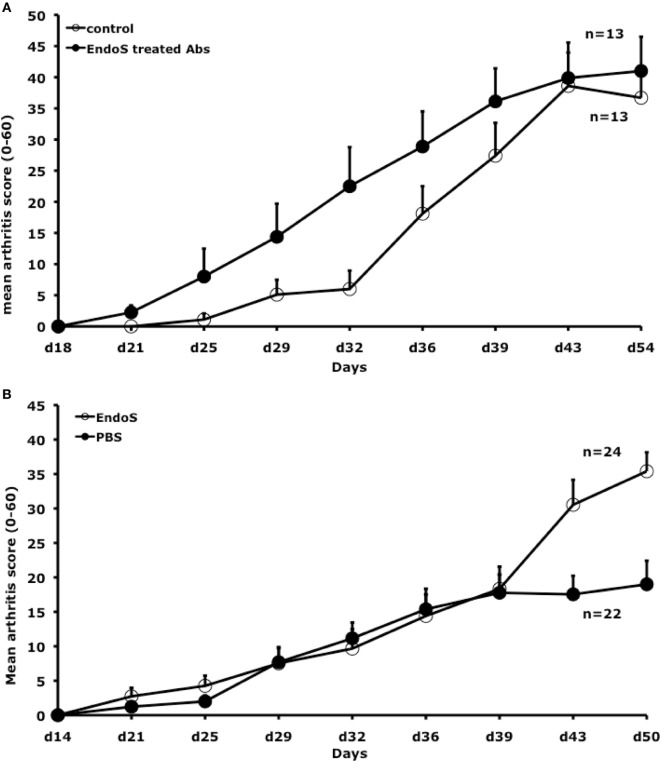
Neither endo-β-*N*-acetylglucosaminidase (EndoS) treated IgG nor EndoS inhibits collagen-induced arthritis. **(A)** Mean arthritis score in male (BALB/c × B10.Q) F1 mice (*n* = 26, 13 mice/group) is shown. Arthritis was induced with rat CII/CFA immunization on day 0 and treated i.v. with 2 mg of EndoS-hydrolyzed antibodies (1 mg each of M2139 and CIIC1) or PBS on days 14, 21, 28, and 35. Error bars indicate ± SEM. **(B)** Mean arthritis score in male (BALB/c × B10.Q) F1 mice (*n* = 46, 22–24 mice/group) were shown. Arthritis was induced with rat CII/CFA immunization on day 0 and treated i.v. with 50 µg of EndoS or PBS on days 14, 21, 28, and 35. Error bars indicate ± SEM.

Previously, we reported RF-like activity of one of the mAbs (CIIC1) present in the arthritogenic cocktail (40); hence, it might be possible that the observed inhibition of arthritis by EndoS hydrolyzed IgG might be due to this property of CIIC1 antibodies. However, we did not observe any difference in CIIC1 mAb binding activity to EndoS hydrolyzed and unhydrolyzed IgG (Figure 8), thereby ruling out the contribution of RF-like activity of CIIC1 in the inhibition of antibody-initiated inflammation. Though EndoS is highly potent in inhibiting antibody-mediated inflammation, we did not find any arthritis inhibitory activity of EndoS-hydrolyzed IgG (Figure 9A) or direct injection of the enzyme (Figure 9B) using collagen-induced arthritis model.

## Discussion

Cleavage of the Fc glycan by the streptococcal enzyme EndoS leads to severely impaired effector functions. Here, we show that very small amounts of EndoS injected *in vivo* in the mouse blocks the development of CAIA. Based on our findings, we propose that EndoS and enzyme treated IgG disrupted larger IC lattice formation through destabilization of all the Fc effector functions including Fc–Fc interactions between pathogenic and deglycosylated Fc domains but without involving changes in C1q and C3b binding, leading to attenuation of joint inflammation.

It has earlier been reported that treatment of mice with higher doses (10–100 µg) of EndoS can suppress many antibody-mediated autoimmune disease models [thrombocytopenic purpura (23), arthritis (22), glomerulonephritis (45, 46), encephalomyelitis (47), hemolytic anemia (48), and epidermolysis bullosa acquisita (49)]. By contrast, we found that EndoS treatment of CIA could not affect the disease development although treatment was done with high EndoS doses (200 µg). However, CIA is a complex disease in which not only pathogenic antibodies are of importance and in addition, initiation and duration of arthritogenic antibody synthesis in the CIA model is likely to be highly variable. By contrast, in the CAIA model, we found that EndoS had a potent therapeutic effect. This was dependent on a strict time window indicating that EndoS is effective during the formation of ICs and also after 48 h of antibody transfer. In addition, we found that though the EndoS was effective in very low doses on CAIA, the same treatment protocols, with EndoS or with hydrolyzed IgG containing small amounts of EndoS had no effect on CIA.

Binding of the arthritogenic antibodies to the cartilage matrix happens within minutes after intravenous injection as detected using positron emission tomography (50) and the subsequent formation of ICs on the joint surface is likely to be the major factor leading to the clinically apparent inflammation and arthritis. In the process of local IC formation, Fc:Fc interactions play an important role (51), and the specific glycans present in the CH2 domain of IgG might have a vital function in this process. Since the suppression seems to be mediated through more acute effects during the binding of antibodies to the cartilage, we hypothesized this might be due to the instability of ICs formed within the target tissue, the articular joints (as illustrated in Figure 7F). Specific IgG glycan hydrolysis alters both murine and human IgG–FcγR interactions (21, 22) and, removal of outer-arm sugar residues affects the thermal stability and functionality of the CH2 domains of IgGs (52). However, the length and nature of residual carbohydrate structures could also affect Fc:Fc interactions and thereby IC formation, complement binding and FcR binding capacities. It is of interest to note that EndoS hydrolysis of IgG does not influence the interactions with the neonatal FcR; hence, overall circulation time of antibodies should be unaffected. However, after EndoS treatment of IgG binding to the other FcRs is affected to varying extent *in vitro* (21), which indeed could attenuate cell-mediated pathology and possibly also clearance of dead cells. Interestingly, it has been shown that IgG maintains several of its FcR-dependent activities with just a mono- or disaccharides present on the Fc glycan *in vivo* (53).

Anti-inflammatory property of terminal sialic acids present on IgG-Fc was demonstrated earlier (11). C-type lectin receptor SIGN-R1 (CD209) expressed on macrophages in the splenic marginal zone is required for recognition of such sialic acids (54), which results in the production of IL-33 and expansion of IL-4-producing basophils promoting increased expression of the inhibitory FcγRIIb on effector macrophages leading to attenuation of inflammation (55). However, in this study, splenectomy did not alter the inhibitory capacity of EndoS-hydrolyzed IgG suggesting involvement of other mechanisms than the SIGN-R1 pathway.

Analysis of immunohistochemical staining of the EndoS and enzyme-hydrolyzed and unhydrolyzed antibody-injected joints for complement activation has led to the conclusion that the presence of small amounts of EndoS and EndoS-hydrolyzed IgG indeed decreased the formation of larger IC *in situ*, most likely by *in vivo* cleaving of IgG sugars and through the disturbance of Fc:Fc-interactions. Significance of Fc:Fc interactions in precipitation (56) and formation of insoluble ICs (51) are early wisdoms. In addition, dinitrophenol specific non-precipitating antibodies were shown to inhibit as well as solubilize IC between antigen and precipitating antibodies (57) that might involve Fc:Fc interactions. Later, structural evidence for such interactions involving the glycosylation loop of one Fc-fragment dimer binding to the CH2–CH3 interface of another Fc fragment has been demonstrated (58). Although oligosaccharides have been reported not to be involved in direct contacts with symmetry-related molecules (58), their interactions with the protein moiety in the IgG–Fc region could very well affect the reciprocal influences on conformation (8). Recent studies also showed that reduced Fc/FcγR interactions through Fc deglycosylation by treatment with EndoS led to improvement of imaging specificity (59) as well as reduction in IC-mediated neutrophil activation (60). Importance of the size of the IC on its effector functions possibly by changes in its interactions with FcγRs has been documented (61).

Our present data clearly demonstrate that EndoS is the causal factor in preventing antibody-mediated inflammation. As shown in Figure 5, 0.1 µg of EndoS injected *in vivo* was capable of specifically cleaving most of the carbohydrates present on all the IgG subclasses, whereas EndoS at 1 µg concentration cleaved all the carbohydrates present in the Fc region of IgG. After intravenous injection, antibodies are present in the circulation for at least 14 days, and the binding of antibodies to the cartilage surface is a dynamic process; hence, it is plausible that the small amounts of EndoS is sufficient to deglycosylate at least part of the injected antibodies, which in turn through destabilization of all the Fc effector functions including Fc–Fc interactions between glycosylated and deglycosylated antibodies could lead to disruption of the formation of larger CII-specific antibody immune-complexes on the joints.

*Streptococcus pyogenes* secretes several enzymes and proteins that bind and modulate the functions of Igs as a part of its strategy for evading the immune system (62). Disruption of the development of larger IC lattices by EndoS and enzyme-cleaved IgG could very well be one such strategy. Conversely, antibodies as a constituent of ICs play an important role in promoting various inflammatory processes. Neutrophils play a vital part during this process, and sequential complement fixation generating C5a and direct engagement of Fcγ receptors are needed to initiate and sustain such neutrophil recruitment *in vivo* and subsequent inflammation (63). Bidirectional regulation of C5aR and FcγRs, which could significantly influence effector functions, was reported earlier (64). At the same time, IgG_1_ containing ICs could also suppress C5a-dependent inflammatory response. This suppression is dependent on high galactosylation of IgG N-glycans, because it promotes the association between FcγRIIB and dectin-1 (65).

In conclusion, very small amounts of residual EndoS present along with the glycan hydrolyzed IgG, but not the IgG with truncated sugars *per se*, inhibits arthritis. It highlights the extreme potency of EndoS *in vivo* and that the potency of EndoS is confined to a local effect on the formation and functions of ICs.

## Ethics Statement

This study was carried out in accordance with the recommendations of “Regional ethical committee, Malmö-Lund region” and Regional ethical committee Stockholm (North), Sweden. The protocol was approved by the “Regional ethical committee, Malmö-Lund region” and Regional ethical committee Stockholm (North), Sweden.

## Author Contributions

KSN participated in the conception and design of the study, acquisition, analysis, and interpretation of the data, and wrote the manuscript. MC, KH, SL, AC, and BX participated in the acquisition, analysis, and interpretation of the data, manuscript preparation, and final approval. RZ participated in the design of the study, manuscript preparation, and final approval. MR, AB, and CK participated in the design of the study, interpretation of the data, manuscript preparation, and final approval. RH participated in the conception and design of the study, interpretation of the data, manuscript preparation, and final approval.

## Conflict of Interest Statement

Hansa Medical AB (HMAB) holds patents for using EndoS as a treatment for antibody-mediated diseases. MC is listed as one of the inventors on these applications and has a royalty and consultancy agreement with HMAB. Genovis AB holds patents for the biotechnological use of EndoS on which MC is listed as an inventor.
